# Prognostic significance of serum miR-18a-5p in severe COVID-19 Egyptian patients

**DOI:** 10.1186/s43141-023-00565-y

**Published:** 2023-11-13

**Authors:** Riham Abdel-Hamid Haroun, Waleed H. Osman, Asmaa M. Eessa

**Affiliations:** 1 Department of Biochemistry, Faculty of Science, Ain Shams University, Cairo, Egypt; 2 Department of Geriatric Medicine and Gerontology, Faculty of Medicine, Port-Said University, Port-Said, Egypt

**Keywords:** SARS-CoV-2 infection, COVID-19, MicroRNAs, hsa-mir-18a-5p, Intensive care unit

## Abstract

**Background:**

The identification of miRNAs as well as characterization of miRNA-mRNA interactions in SARS-CoV-2 infection is important to understand their role in disease pathogenesis. Therefore the aim of the present study was to measure the expression levels of hsa-mir-18a-5p in the sera of severe COVID-19 Egyptian patients admitted to ICU to investigate its roles in the pathogenesis and severity of COVID-19 disease.

**Methods:**

A total of 180 unvaccinated severe COVID-19 patients were enrolled in our study. Besides the routine laboratory work, the expression level of hsa-mir-18a-5p was done using reverse transcription quantitative real-time PCR (RTqPCR) technique. Also, target genes of hsa-mir-18a-5p were explored by using online bioinformatics databases.

**Results:**

The expression level of hsa-mir-18a-5p decreased in nonsurvival severe COVID-19 patients (0.38 ± 0.26) when compared to the survival ones (0.84 ± 0.23). While as a prognostic tool for the prediction of bad prognosis and mortality among severe COVID-19 patients, our results showed that the serum hsa-mir-18a-5p expression level is a good sensitive and specific marker. By using bioinformatics tools, our results revealed that the decreased hsa-mir-18a-5p expression level may have a crucial role in COVID-19 pathogenesis and severity through decreased immunological responses (interpreted as lymphopenia) or increased inflammation (interpreted as increased serum levels of IL-6, CRP, LDH).

**Conclusion:**

Taken together, the decreased expression level of hsa-mir-18a-5p could be a bad prognostic marker and therapeutic overexpression of hsa-mir-18a-5p could be a novel approach in the treatment of COVID-19 disease.

## Introduction

Due to the high prevalence and long incubation periods, the severe acute respiratory syndrome coronavirus-2 (SARS-CoV-2) has infected millions of individuals globally, causing the coronavirus disease 2019 (COVID-19) pandemic [1]. It was first emerged in Wuhan, China in December 2019 and declared a global pandemic by WHO on 11 March 2020 [2, 3]. It has become a catastrophic public health crisis affecting many people as of January 09, 2023, there have been 668,820,532 confirmed cases, and 6,714,775 deaths were reported in more than 229 countries [4]. The clinical presentation of COVID-19 varies so much from asymptomatic to milder symptoms, including dry cough, fever, myalgia, dyspnea, sore throat, and headache, or even to severe and emergent manifestations including chest pain, confusion, hypoxia, pneumonia, and other complications requiring intensive care unit (ICU) admission and mechanical ventilation [5]. The clinical guidelines of WHO define “severe COVID-19” as patients with clinical signs of pneumonia (fever, cough, dyspnea, and fast breathing) accompanied by one of the following: severe respiratory distress; O2 saturation (SpO2) ≤ 90% in room air; or respiratory rate > 30 breaths/min [6]. Till now, the precise determinants of severe COVID-19 are not known, but it primarily maybe host factors rather than viral genetic mutations [7]. The number of COVID-19 patients is continually increasing worldwide and the management in ICU has become a major challenge; therefore, early recognition of severe forms of COVID-19 is very necessary for triaging of COVID-19 patients [8].

MicroRNAs (miRNAs) are a class of highly conserved endogenous small (18–22 nt) noncoding single-stranded RNA molecules widely found in plants, animals, and some viruses. They have an essential role in post-transcriptional regulation of gene expression by targeting the mRNAs of protein-coding genes [9]. MiR-18a-5p is located at chromosome 13q31.3 and belongs to Mirc1 locus, better known as the miR-17–92 cluster, encodes six miRNAs (miR-17-5p, miR-18a-5p, miR-19a-3p, miR-19b-3p, miR-20a-5p, and miR-92a-3p); which has important roles in cell proliferation and differentiation, inflammation, immunity and immunological process [10, 11]. It was reported that miRNAs have been shown to have a role in viral infections as viruses can induce the up- or downregulation of various host miRNAs to elude the host’s immune system [12]. It was found that miR-18a-5p was reduced in bleomycin-treated pleural mesothelial cells (PMCs) which in turn contributes to epithelial-mesenchymal transition (EMT) of PMCs via upregulation of its target, TGF-β receptor II (TGF-βRII), which mediates signaling leading to sub-pleural pulmonary fibrosis [13]. Therefore the aim of the present study was to measure the expression level of hsa-mir-18a-5p in the sera of unvaccinated severe COVID-19 Egyptian patients admitted to ICU and then examine the target genes of has-mir-18a-5p using bioinformatics online tools to investigate its role in the pathogenesis and severity of COVID-19 disease also its correlations with other clinical variables in severe COVID-19 patients.

## Patients and methods

### Ethics statement

The current study was approved by the ethics committee of the Faculty of Medicine, Port-Said University, Egypt (ERN MED (23/04/2020)S.no(5)MED). Informed consent was obtained from all patients.

### Human subjects and data collection

The current study was conducted on 180 unvaccinated severe COVID-19 patients recruited from the isolation hospitals in Port-Said, Egypt. Sputum and throat swab specimens (for qPCR for SARS-Cov-2 RNA test) and blood samples were collected from all patients. Laboratory tests were conducted at admission, including a complete blood count, liver function tests (ALT and AST), kidney function tests (urea and creatinine), CRP, ferritin, IL-6, D-dimer, PCT, and LDH. Also, chest CT scans are made for all patients. The severity of COVID-19 was graded according to Suspected COVID-19 Cases Management in Triage Hospitals by the Ministry of Health and Population of Egypt. Our Severe COVID-19 patients were defined as patients with respiratory distress, resting oxygen saturation ≤ 90%, respiratory failure requiring mechanical ventilation, or failure of other organs requiring ICU admission.

Data from severe patients were collected from the latest laboratory tests prior to physicians making the clinical diagnosis of severe disease. Demographic data, hospitalization time, medical history, oxygen saturation, respiratory rate, oxygen supply, laboratory findings, and thorax tomography of the patients were obtained from the hospital’s electronic information system retrospectively. All patients were treated by meropenem (1 gm/8 h intravenous), levofloxacin (500 mg vial/24 h, intravenous), linezolid (600 mg vial/8 h, intravenous), Enoxaparine calcium (therapeutic dose, subcutaneous), methylprednisolone sodium succinate (1 gm every 24 h for 3 days then 125 mg every 12 h), tocilizumab (if needed, 8 mg/kg), and remdesivir (loading dose 400 mg first day, then 200 mg for 5 days).

### Determination of serum miR-18a expression level by RT-qPCR

#### MiRNA extraction and cDNA preparation

The miRNA was extracted from the sera of all patients using miRNeasy Mini kit (cat # 217004, Qiagen, USA) according to the manufacturer’s instructions. The purity and the concentration of the purified miRNA were detected using spectrophotometer nano-drop (Quawell, Q-500, Scribner, USA) and stored at − 80 °C till further assessments. To synthesize cDNA, miRNA was reverse transcribed using MiScript II reverse transcription kit (cat # 218160, Qiagen, USA) according to manufacturer’s instructions and stored at − 20 °C till performing qPCR.

#### Quantitative real-time PCR (qPCR)

Quantitative real-time PCR was performed using miScript primer assay (cat # 218300, Qiagen, USA) for miR-18a (Hs_miR-18a_2 miScript Primer Assay, MS00031514); the reaction was carried out using MiScript SYBR Green PCR kit (cat # 218073, Qiagen, USA). Also, RNU6–2 (Hs_RNU6-2_11 miScript Primer Assay, MS00033740) was used as an endogenous control to normalize the expression levels of the investigated miRNAs; the primer sequences are listed in Table 1. The qPCR cycling conditions were as follows: 95 °C for 10 min, followed by 40 cycles of 95 °C for 15 s, 55 °C for 30 s, and 72 °C for 30 s in which fluorescence was acquired and detected by Stratagene Real-time PCR system (Max3005P QPCR system, Stratagene, Agilent biotechnology, USA). The relative expression levels of the investigated miRNAs were evaluated using the 2^−ΔΔCq^ method described by Livak and Schmittgen [14]. A 2-fold increased (≥ 2) or decreased (≤ 0.5) value was considered mRNA overexpression or downregulation, respectively.

**Table 1:** Primer sequences for quantitative RT-PCR analysis

Gene	Primer sequence
miR-18a	5′-UAAGGUGCAUCUAGUGCAGAUAG-3′
RNU6B	5′-CUCGCUUCGGCAGCACAUAUACUAA-3′

### Bioinformatics analysis

To examine the target genes of has-mir-18a-5p, different online databases were used, miRDB (https://mirdb.org/mirdb/index.html), TargetScan (https://www.targetscan.org/vert_80/), DIANA-TarBase(https://dianalab.e-ce.uth.gr/html/diana/web/index.php?r=tarbasev8%2Findex/), miRwalk (http://mirwalk.umm.uni-heidelberg.de/), miRNet (https://www.mirnet.ca/miRNet/home.xhtml/).

### Statistical analysis

Statistical analysis was performed using IBM SPSS software (version 23.0; IBM Corp., Armonk, NY, USA), and data were presented as means ± S.D. One-way ANOVA was used to determine statistically significant differences between group’s means and Pearson’s correlation coefficient was used to determine significant correlations of serum has-mir-18a-5p expression level with other clinical parameters. The receiver operating characteristic curve (ROC curve) was used to calculate the area under the curve (AUC), sensitivity, and specificity of serum has-mir-18a-5p expression level as a biomarker for the detection of bad prognosis and deterioration of severe COVID-19 disease. The criterion for significance was *p* < 0.05.

## Results

### Demographic and biochemical data of COVID-19 patients

The present study included 180 severe COVID-19 patients; 108 males and 72 females; with mean age 67.4 ± 9.6 years; oxygen saturation 86.32 ± 4.05%; respiratory rate 26.83 ± 3.17 cycle/min; and 74 (41.1%) patients died during hospitalization; the clinical and biological data of severe COVID-19 patients are summarized in Table 1. The results of current study revealed a highly significant (*p* < 0.001) increase in the levels of CRP (76.29 ± 25.50), IL-6 (383.49 ± 213.84), PCT (0.82 ± 0.35), and LDH (480.43 ± 96.08) in the sera of nonsurvival severe COVID-19 patients when compared to survival severe COVID-19 patients (59.81 ± 16.69; 63.75 ± 44.71; 0.19 ± 0.14 and 273.54 ± 99.68; respectively). Also, the blood neutrophils percentage (78.61 ± 6.13) was significantly (*p* < 0.001) increased while lymphocytes percentage (11.63 ± 5.35) was significantly (*p* < 0.001) decreased among nonsurvival severe COVID-19 patients as compared with survival severe COVID-19 patients (69.12 ± 14.62 and 19.91 ± 4.94; respectively); as shown in Table 2.

**Table 2:** Clinicopathological characteristics and CT findings of severe COVID-19 patients

Variable		
Group	Severe COVID-19 patients	
	Survivals (*n* = 106) (Mean ± SD)	Non-survivals (*n* = 74) (Mean ± SD)
Age (years)	66.3 ± 10.1	68.6 ± 8.9
Gender (*n* (%))		
Male	59 (55.7%)	49 (66.2%)
Female	47 (44.3%)	25 (33.8%)
Urea (mg/dl)	35.42 ± 9.83	40.11 ± 9.55
Creatinine (mg/dl)	1.06 ± 0.27	1.15 ± 0.31
AST (U/L)	35.30 ± 13.94	42.28 ± 21.88*^a^
ALT (U/L)	34.67 ± 19.23	36.21 ± 20.89
WBCs (10^3/μl)	12.53 ± 4.61	13.98 ± 5.87
Neutrophils (%)	69.12 ± 14.62	78.61 ± 6.13*^a^
Lymphocytes (%)	19.91 ± 4.94	11.63 ± 5.35 *^a^
CRP (mg/dl)	59.81 ± 16.69	76.29 ± 25.50**^a^
Ferritin (ng/ml)	425.97 ± 142.49	453.25 ± 161.11
IL-6 (pg/dl)	63.75 ± 44.71	383.49 ± 213.84**^a^
D-dimer (mg/L)	1.99 ± 0.34	2.24 ± 0.96
PCT (ng/ml)	0.19 ± 0.14	0.82 ± 0.35**^a^
LDH (U/L)	273.54 ± 99.68	480.43 ± 96.08**^a^
O_2_ saturation (%)	91.47 ± 3.91	90.18 ± 4.0
Respiratory rate (cycle/min)	26.97 ± 3.04	26.86 ± 2.87
Oxygen supply (*n* (%))		
CPAP	49 (46.2%)	42 (56.7%)
NRM	57 (53.8%)	32 (43.3%)
Chest CT findings (*n* (%))		
CORADs 4	28 (26.4%)	35 (47.3%)
CORADs 5	78 (73.6%)	39 (52.7%)

*Significant at *p* value < 0.05

**Highly significant at *p* value < 0.001

^a^Significant difference versus survival severe COVID-19 (control) group

### Serum has-mir-18a-5p expression level and receiver operating characteristic (ROC) curves analysis

**Fig. 1:**
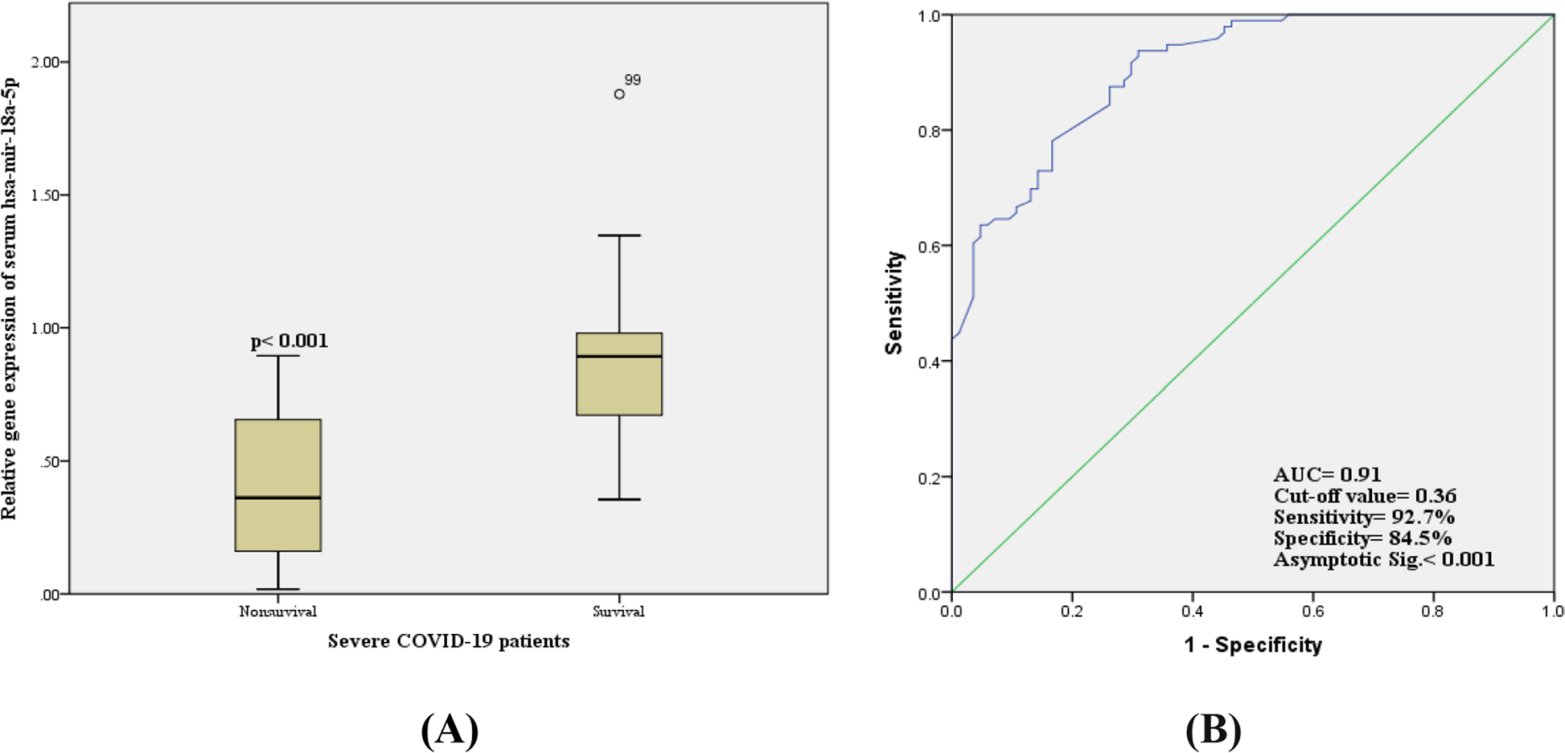
Serum hsa-mir-18a-5p expression level among severe COVID-19 patients: **A** relative gene expression of hsa-mir-18a-5p in nonsurvival and survival severe COVID-19 patients. **B** ROC curve of serum hsa-mir-18a-5p expression level in discrimination between survival and non-survival severe COVID-19 patients

Our results showed that serum has-mir-18a-5p had a differential expression pattern, as it was found to be highly significantly (*p* < 0.001) decreased in nonsurvival COVID-19 patients (0.38 ± 0.26) when compared to the survival ones (0.84 ± 0.23); as shown in Fig. 1A. The sensitivity and specificity as biomarker of serum has-mir-18a-5p expression level for the prediction of bad prognosis and mortality among severe COVID-19 patients were evaluated by using ROC curve analysis. Our results showed that it is a good biomarker that could predict a bad prognosis of severe COVID-19 patients, with AUC 0.91, 92.7% sensitivity, and 84.5% specificity; as shown in Fig. 1B.

### Correlation of serum has-mir-18a-5p expression level with clinical variables in severe COVID-19 patients

Data recorded in Table 3 shows the correlation matrix of serum has-mir-18a-5p expression level with the different clinical parameters in this study. It was found that it was highly significantly positively correlated (*p* < 0.001) with lymphocyte percentage, while it was highly significantly negatively correlated (*p* < 0.001) with neutrophils percentage, CRP, IL-6, PCT, and LDH; as shown in Table 3.

**Table 3:** Correlations of serum has-mir-18a-5p with different parameters among severe COVID-19 patients

miR		
Variable	has-mir-18a-5p	
	*r*	*p* value
Urea (mg/dl)	− 0.14	0.07
Creatinine (mg/dl)	0.09	0.223
AST (U/L)	0.03	0.63
ALT (U/L)	− 0.13	0.08
WBCs (10^3/μl)	− 0.21	0.79
Neutrophils (%)	− **0.27**	0.000**
Lymphocytes (%)	**0.29**	0.000**
CRP (mg/dl)	− **0.31**	0.000**
Ferritin (ng/ml)	− 0.06	0.39
IL-6 (pg/ml)	− **0.56**	0.000**
D-dimer (mg/L)	0.001	0.99
PCT (ng/ml)	− **0.23**	0.002**
LDH (U/L)	− **0.54**	0.000**

*Significant at *p* value < 0.05

**Highly significant at *p* value < 0.001

### Bioinformatics analysis

**Fig. 2:**
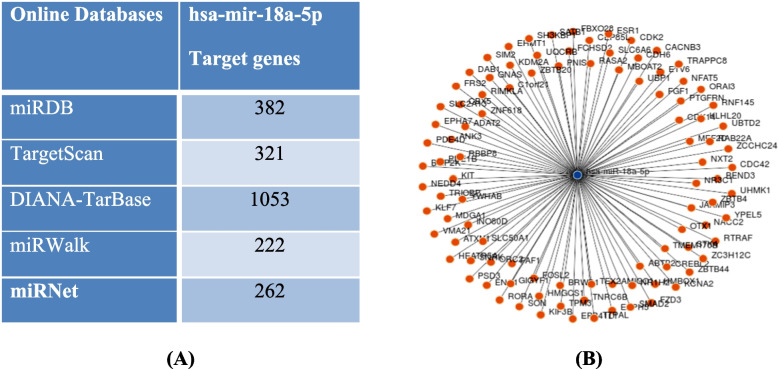
Network analysis of hsa-mir-18a-5p target gene networks: **A** numbers of target genes of hsa-mir-18a-5p. **B** Visualization of target genes of hsa-mir-18a-5p using miRWalk online database

**Fig. 3:**
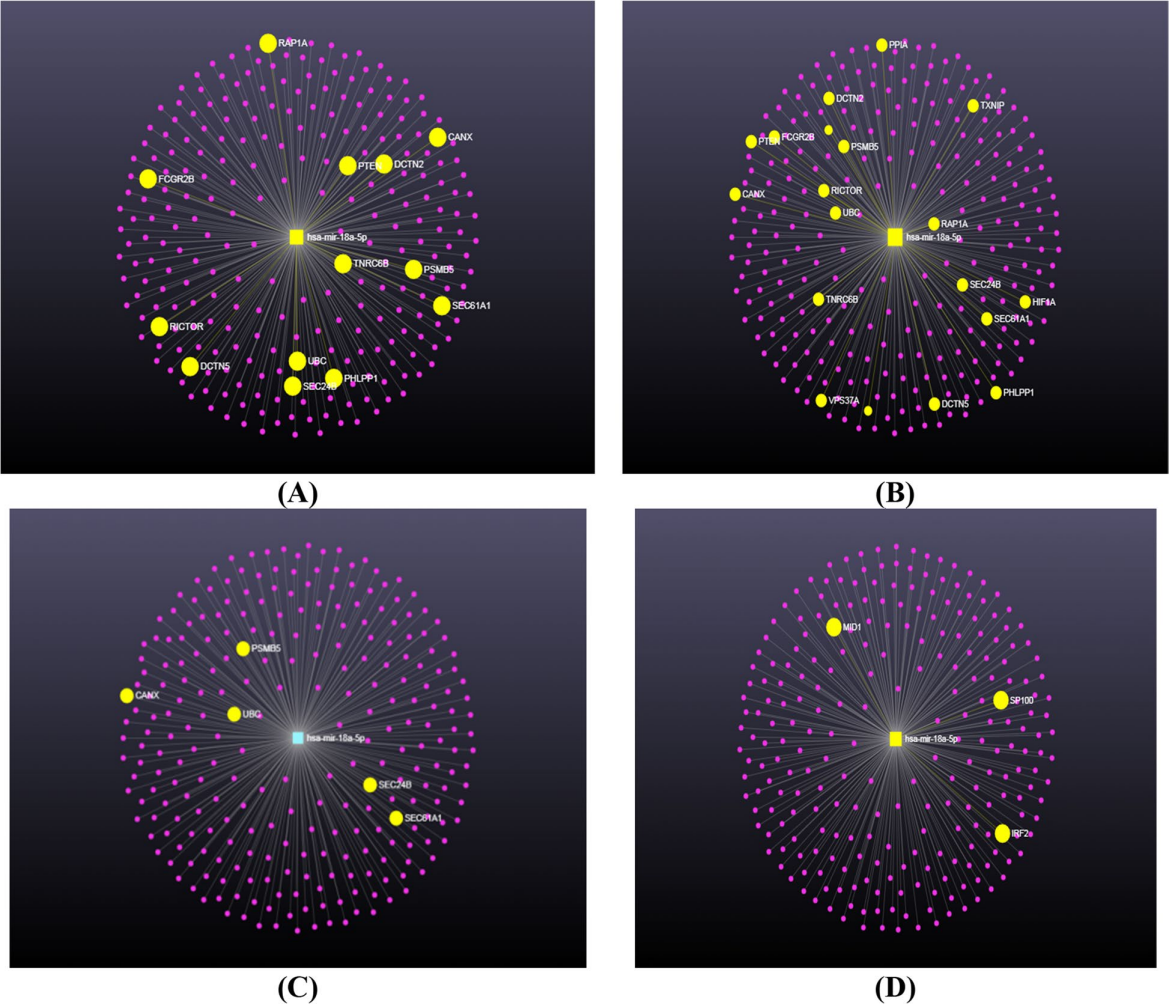
Target genes of hsa-mir-18a-5p in immunological responses: **A** adaptive immune system. **B** Signaling events of B cell receptor (BCR). **C** Class I MHC mediated antigen processing and presentation. **D** Interferon gamma signaling

**Fig. 4:**
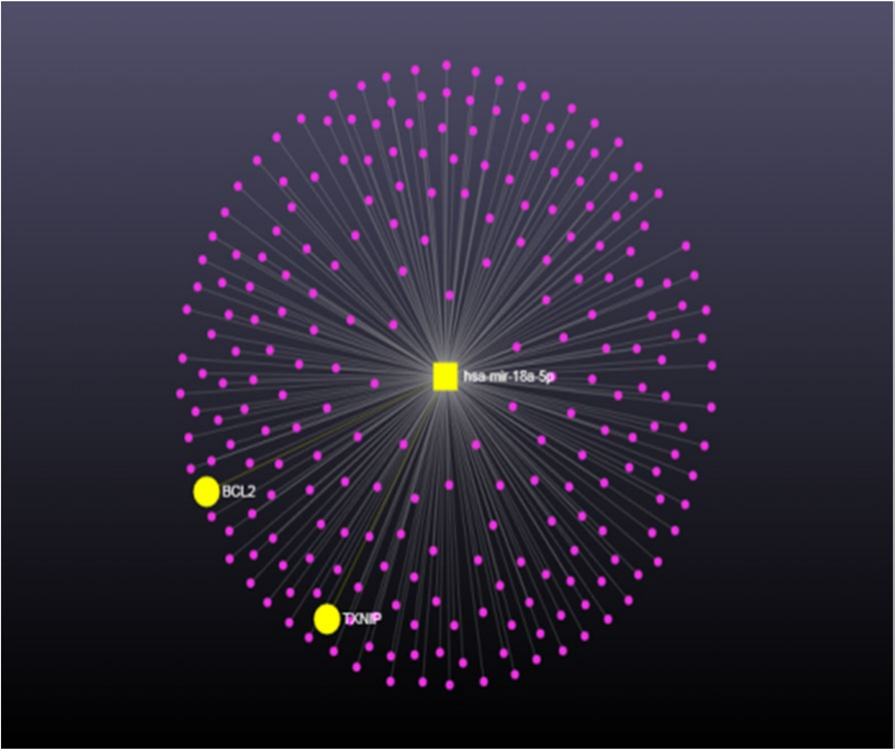
Target genes of hsa-mir-18a-5p in inflammation

Different online databases were used to investigate the target genes of hsa-mir-18a5p. Different numbers of target genes were obtained, miRDB (382), TargetScan (321), DIANA-TarBase (1053), miRwalk (222), and miRNet (262); as shown in Fig. 2A. To visualize the target genes of has-mir-18a-5p as a figure, authors used miRwalk database (Fig. 2B), while to predict its possible roles or pathways in the COVID-19 pathogenesis and severity, miRNet database was used. According to miRNet database, our results showed that hsa-mir-18a5p has a role in the adaptive immune system by targeting CANX, FCGR2B, PSMB5, PTEN, RAP1A,UBC, EC24B, DCTN2, TNRC6B, PHLPP1, SEC61A1, DCTN5, and RICTOR genes; as shown in Fig. 3A. Also it has signaling events of B cell receptor (BCR) by targeting PSMB5, PTEN, UBC, TNRC6B, PHLPP1, and RICTOR genes; as shown in Fig. 3B. Moreover, hsa-mir-18a5p mediated Class I MHC antigen processing and presentation through CANX, PSMB5, UBC, SEC24B, and SEC61A1 genes; as shown in Fig. 3C. Furthermore, it has a crucial role in interferon gamma signaling by targeting IRF2, MID1, and SP100 genes; as shown in Fig. 3D. Other than the immunological responses, hsa-mir-18a5p has a role in inflammation (by targeting BCL2 and TXNIP genes; as shown in Fig. 4).

## Discussion

It is well known that miRNAs play an important role in the posttranscriptional control of gene expression that is dysregulated in different physiological pathophysiological processes, such as metabolism, growth, cell differentiation and development, apoptosis, inflammation, and cell signaling [15]. Besides, the role of miRNAs in the pathogenesis of lung disease has been recognized, as Dakhlallah et al. [16] reported that downregulation of miR-17∼92 contributes to the pathogenesis of pulmonary fibrosis, and also miR-18a levels are extraordinarily decreased in the lung of human IPF patients [16, 17]. However, the mechanisms involved in the regulatory effects of miRNA in pulmonary fibrosis have not been revealed. Therefore, we aimed to measure the expression levels of has-mir-18a-5p in the sera of unvaccinated severe COVID-19 Egyptian patients admitted to ICU to investigate its roles in the pathogenesis and severity of COVID-19 disease also its correlations with other clinical variables in severe COVID-19 patients. In our previous work, we measured the levels of serum IP-10, SAA, and sialic acid and circulating plasma has-mir-155-5p in positive COVID-19 patients to explore their clinical values and significance in discrimination between moderate and severe COVID-19 infection and predicting the severity and prognosis of COVID-19 disease [18–21]. Here, our results revealed that the expression level of serum has-mir-18a-5p was significantly decreased in nonsurvival COVID-19 patients when compared to the survival ones (Fig. 1A). As consistent with our results; Li et al. found that the level of miR-18a is down-expressed in the peripheral blood from human patients with COVID-19 [22], also other several studies reported miR-18a down expression in asthma patients [23, 24]. Moreover, Ventura et al. [25] reported that the loss-of-function of the miR-17-92 cluster resulted in smaller embryos and immediate postnatal death of all animals due to severely hypoplastic lungs of mice lacking miR-17-92, indicating the vital role of miR-18a in the proper function of lungs. Several studies reported the diagnostic and prognostic significance of serum mir-18a-5p [26–28]. Therefore, the current study also aimed to elaborate and assess the potential role of serum mir-18a-5p as a prognostic biomarker for the prediction of bad prognosis and mortality among severe COVID-19 patients. Our results showed that it is a good biomarker that could predict the bad prognosis of severe COVID-19 patients, with AUC 0.91, 92.7% sensitivity, and 84.5% specificity (Fig. 1B).

The innate immune response to SARS-CoV-2 Antiviral innate immunity has several humoral components, including mannose-binding lectin, interferons, chemokines, B lymphocytes, natural killer cells, and other innate lymphoid cells (ILCs) and gamma delta T cells, which generally limit the spread of viral infection by cytotoxic action on target cells, cytokine production, and induction of an adaptive response [29]. By using bioinformatics online tools, it was found that hsa-mir-18a-5p targets many genes, which may be involved in many pathways such as immunological responses (Fig. 3A–D). Therefore, as the expression level of hsa-mir-18a-5p decreased, these immunological responses such as adaptive immune responses (Fig. 3A), signaling events of B cell receptor (Fig. 3B), class I MHC mediated antigen processing and presentation (Fig. 3C) and interferon-gamma signaling (Fig. 3D) were decreased. These observations are in line with the relative lymphopenia reported in severe COVID-19 and also in our patients. It is well known that hsa-mir-18a-5p regulates the immunological responses, especially in respiratory diseases as in a study about influenza A, miR-18a-5p was found to be involved in the regulation of the pulmonary innate immune response [30]. Moreover, several viruses, including SARS-CoV-2 have been reported to enhance TGF-β signaling, which is known to induce fibrosis and suppress adaptive immunity through a modulation of TGF-β signaling, via the surface receptors and canonical SMAD and MAPK pathways regulated by hsa-mir-18a-5p regulating adaptive immune responses [31].

The pathogenesis of COVID-19 is complex, but it can be conceptually described using typical models for the three main pathological processes associated with inflammation—local manifestations of classical general (canonical) inflammation, acute systemic inflammation, and chronic systemic inflammation of low intensity [32]. Our bioinformatics analysis results showed that hsa-mir-18a5p has an inhibitory effect on inflammation (Fig. 4), which in turn may play crucial roles in COVID-19 pathogenesis and severity.

As consistent with our results, it was indicated that miR-18a-5p mimic significantly reduced inflammatory factors including IL-6, IL-8, IL-1β, and tumor necrosis factor (TNF)-α release, decreased the degranulation rate and histamine release rate of cells [33], this may explain the increased serum level of IL-6 in our severe COVID-19 patients, especially the nonsurvival ones. Moreover, Geng et al. [34] observed that has-mir-18a-5p upregulation prevents endothelial-mesenchymal transition and cardiac fibrosis induced by high glucose concentration, by targeting NOTCH2 gene, which regulates cellular phenotype. Our results also revealed that has-mir-18a-5p showed very significant correlations with the other parameters by Pearson correlation analysis, which suggested that it was a significant factor associated with the severity of patients with COVID-19.

There are some limitations that need to be addressed regarding the present study. First of all, the population was only from Egypt, which reduces the possibility of confounding by ethnicity; therefore, these results should be interpreted with caution. Second, given the limited size of the study and additional large-scale studies are needed to confirm this finding. Finally, serum levels or gene expression levels of target genes of has-mir-18a-5p should be measured concurrently with has-mir-18a-5p levels in the same patients’ samples to ensure their interactions.

## Conclusion

Finally, by using survival severe COVID-19 patients as a control group our results showed that the expression level of hsa-mir-18a-5p was significantly decreased among the nonsurvival severe COVID-19 patients. While as a prognostic tool for the prediction of bad prognosis and mortality among severe COVID-19 patients, our results showed that the serum hsa-mir-18a-5p expression level is a good sensitive and specific marker. By using bioinformatics tools, our results revealed that the decreased hsa-mir-18a-5p expression level may have a crucial role in COVID-19 pathogenesis and severity through decreased immunological responses (interpreted as lymphopenia) or increased inflammation (interpreted as increased serum levels of IL-6, CRP, LDH). This provides proof of concept that the therapeutic overexpression of hsa-mir-18a-5p could be a novel approach in the treatment of COVID-19 disease.

## Data Availability

All data generated or analyzed during this study are included in this published article.

## Abbreviations

AUC: Area under the curve
BCL2: B cell lymphoma 2
CANX: Calnexin
COVID-19: Coronavirus disease 2019
CRP: C-reactive protein
DCTN2: Dynactin subunit 2
DCTN5: Dynactin 5 (p25)
FCGR2B: Fc fragment of IgG receptor IIb
ICU: Intensive care unit
IL-6: Interleukin 6
IRF2: Interferon regulatory factor 2
LDH: Lactate dehydrogenase
MAPK: Mitogen-activated protein kinase
MID1: Midline-1
miRNAs: MicroRNAs
NOTCH2: Neurogenic locus notch homolog protein 2
PCT: Procalcitonin
PHLPP1: PH domain and leucine-rich repeat protein phosphatases
PSMB5: Proteasome subunit beta type-5
PTEN: Phosphatase and tensin homolog
RAP1A: Ras-related protein Rap-1A
RICTOR: Rapamycin-insensitive companion of mammalian target of rapamycin
ROC curve: Receiver Operating Characteristic curve
SARS-CoV-2: Severe acute respiratory syndrome coronavirus-2
SEC24B: SEC24 Homolog B
SEC61A1: SEC61 Translocon Subunit Alpha 1
SP100: Space reactor prototype
SpO2: O2 saturation
TNRC6B: Trinucleotide repeat-containing gene 6B protein
TXNIP: Thioredoxin-interacting protein
UBC: Ubiquitin C
WHO: World Health Organization
